# The Diet of the Insane

**Published:** 1863-04

**Authors:** Henry M'Cormac

**Affiliations:** Belfast.


### Art. II.—THE DIET OF THE INSANE.

By Henry McCormac, M.D., Belfast.

The regimen, as to food, of the insane is important in more ways than one. It is important in respect of the preservation of their bodily health and strength. It is important as regards their psychical health. There can be no difference of opinion about the first; and if we consider the directly pleasurable influences springing from well-directed appeals to the organ of taste, the influence of the great principle of substitution in the treatment of the insane, even in the matter of foods and drinks, there will be just as little difference of opinion about the latter.

I think that the dietary of the sick in mind should not be inferior to the dietary of the attendants on the insane. I think it would be well if both could commonly dine at the same table. A superior dietary, and more especially a varied dietary, I maintain, is wholly beneficial in the treatment of the insane. I would not have the food of two successive days, much more the food of all the days of the year, entirely alike. In this respect we should imitate the diversity that subsists out of doors, where an inferior, because a varied, dietary is found preferable to a dietary that is superior *per se*, but which is otherwise unvaried.

It costs no more, I assert, to have a good food regimen than a poor and inefficient one. Nay, it will be found cheaper, cheaper both in the first instance and in the last. For man's frame must be supplied with certain essentials, and it only costs a little expenditure of thought and feeling to vary these essentials, day by day, and to offer them in an agreeable, instead of a mean and sordid form.

I do not wish to say that a profuse dietary is the best. What I mean to say is, that it should be varied, agreeable, and attractively served. These last conditions being constantly held in view, there will be a saving—as in the article of animal food, for example, of which about four ounces, with proper condiments, vegetable and cereal accompaniments, will then, I conceive, prove sufficient. A pound of meat and a pound of bread, indeed, must be looked upon as the essential food-staple, the foundations of the animal man. But if only the food-equivalent be administered, it is not absolutely essential that it should be in this form. A little animal food, with a judicious alternation of vegetables, grains, and even fruit, when available, after the fashion of French cookery, would be found preferable to the wedges of plain meat of the English asylums, and very much so to the potatoes and sordid broth of the Irish asylums.

There should be some sumptuosity in the manner of catering for the insane. Glass and porcelain are happily cheap these days. It would cost little to have the one of varied forms and colours, and the other decked as with fruit and flowers. The arts of photography and polychromy are now so advanced as to furnish cheap and abundant resources in these respects; and both would lend their cheering aid in rescuing, to however limited an extent, the afflicted from the monotonous tyranny of their morbid trains of thought.

There might also be some pomp and circumstance in the attendance. A corps of waiters, male and female, might be organized from among the insane. And it might be so arranged, by means of harmless appeals to their vanity and their palate, as to render this office rather a coveted distinction than otherwise.

The band should be in attendance at meal-hours, so as with a judicious alternation of sacred and secular music, to gladden and impress the sufferers. Almost all the insane are capable of receiving agreeable impressions from music, as well as of becoming proficients in it. And it is very desirable that such an innocent, cheap, and effective source of healthy distraction should be turned to the fullest account.

The food of the insane is a matter of so great importance, that I think it should prove the subject of a special commission, with a view to the compulsory adoption of an improved varied dietary in all asylums—English, Irish, and Scottish. The seasons of the year, and the capabilities of the locality, would have to be considered somewhat; but the diet should always be good. Some sort of drink—sweet ale or wine, ought to prove an ingredient in the daily fare. If regimen have any influence, and who can deny that it has influence, a superior and varied dietary, by aiding in the grand result of furthering the happiness and the return to mental sanity, would prove cheaper every way than the sordid and dreary regimen with which we are to some extent content to subject the insane.

The following is the dietary of the Belfast District Hospital for the Insane :—

BREAKFAST.

| | | |
|---|---|---|
| One quart stirabout (made with 8 oz. fine, or 7 oz. coarse meal) and three-fourths of a pint mixed milk | Males. | Every morning. |
| One-and-a-half pint stirabout and one half-pint mixed milk | Females. | |

DINNER.

| | | |
|---|---|---|
| One half-pound loaf bread (or 3 lbs. potatoes), 6 oz. solid meat, and one pint soup | Males & Females. | 3 days a week. |
| One half-pound loaf bread (or 3 lbs. potatoes) and one quart soup (made with ox-heads and bones cut out of meat, vegetables, oatmeal, barley, pease, &c.) | Males. | 3 days a week. |
| One half-pound loaf bread (or 3 lbs. potatoes), and one-and-half pint soup. | Females. | |
| Three-fourths of a pound loaf bread (or 3½ lbs. potatoes) and one pint mixed milk | Males. | 1 day a week. |
| One half-pound loaf bread (or 3 lbs. potatoes) and one pint mixed milk. | Females. | |

SUPPER.

| | | |
|---|---|---|
| One half-pound loaf bread and three-fourths of a pint of new milk | Males. | Summer 6 months. |
| One half-pound loaf bread and one half-pint new milk | Females. | |
| One quart stirabout and three-fourths of a pint new milk | Males. | Winter 6 months. |
| One-and-half pint stirabout and one half-pint new milk. | Females. | |

*** Patients who are actively employed are allowed a portion of meat, in addition to the soup, five instead of three days in the week. When the state of the patient's health requires it, the diet is changed accordingly, and any other substituted that may be considered requisite by the medical officers.

It will be seen that it is more congruous with the habits and mode of life of the patients while in their own poor homes than with that liberal scale of allowance, of which the great importance has been so fully recognised in English asylums for the insane. There is, however, no salt fish in the list, and no salt bacon, articles commonly and very desirably included in the home-food of the Irish poor. The amount is ample, but the food is too little varied; it is, in fact, hardly varied at all, unless the greater or less allowance of potatoes and, conversely, of bread, can be considered variety. I should like each day in the seven to have a somewhat different dietary; I would have both salt fish and salt bacon. I would substitute a meat-stew for the broth, an article, such as it is, of which I should not myself like to partake daily, or indeed to eat of habitually at all. I would prefer to vary the oatmeal porridge by alternating it with messes made of wheaten-meal or Indian-corn meal. I should like to see the introduction of tea, coffee, cocoa, wine, ale and tobacco; and certainly I should prefer whole-meal bread for habitual use to white. The dietary of asylums should everywhere, I think, in these kingdoms be dictated by a Commission, for the nature and amount of human subsistence is not, as many imagine, an arbitrary thing, but should, and indeed, to be efficient, must be graduated by the wants of the living frame. Meanwhile, and provisionally as it were, I would beg to submit the following dietary for the consideration of the gentlemen interested directly in the management of Irish asylums for the insane poor.

*Breakfast.*—On five days, 8 oz. oatmeal, wheaten-meal, or Indian-corn meal, say alternately, made into 1 quart porridge, and 12 oz. milk, plain or mixed. On two days, 1 pint, imperial, of tea or coffee, or cocoa, 5 (or 6) oz. whole-meal bread, and butter ¼ oz. (1 oz. tea, or 2 oz. coffee, and 16 oz. new milk to the gallon.)

*Dinner.*—On two days, 4 oz. fresh meat (boiled, roast, or stewed), 16 oz. potatoes, or 12 oz. of some other vegetable, and 3 oz. (or 4) whole-meal bread. On two days, 12 oz. potatoes, 2 oz. beef, and 1 oz. salt pork, as Irish stew; also 3 oz. (or 4) whole-meal bread, and 1 oz. cheese. On one day a herring, or 4 oz. salt cod or ling, steamed and boned, then made into pie, with in each case 12 oz. potatoes and ¼ oz. butter; also 3 oz. (or 4) whole-meal bread, and 1 oz. cheese. On one day, 3 oz. bacon, or 4 oz. salt pork, and 3 oz. say of peas in 1 pint imperial of soup; also 3 oz. (or 4) whole-meal bread and 1 oz. cheese. And on one day, 1 lb. meat-pie, made in the proportion of 1 oz. salt pork, 3 oz. fresh meat, 2 oz. suet, and 4 oz. flour; also 3 oz. bread and 1 oz. cheese.

*Supper.*—Tea, 1 pint imperial, whole-meal bread 5 oz. (or 6), butter ¼ oz.

The foregoing rations might be more or less modified, according to age, sex, season, health, occupation. Summer and winter, indeed, suggest changes of dietary, in respect of fruit, vegetables, and otherwise. A fruit-pie once or twice a week, when in season, would prove a great boon. Sugar, salt, pepper, are all important in themselves, and as ingredients, according to taste and judgment, in made dishes, so named. Polenta, in shapes, and eaten with treacle, or other simple seasoning, is an excellent occasional article of food. Hominy, or bruised Indian corn, also Indian corn meal, in this country is very cheap and good. And because it is so, some people perversely despise it. Yet, time was, ere Corn Law repeal, when polenta was sold in London for two shillings per pound. In Ireland, just now, a shilling, and two or three pence, will purchase a stone of it. Rice boiled exactly thirty minutes in boiling water, *croquant*, and eaten with milk or treacle, constitutes a very desirable variety of nutriment. Potatoes, before they are brought to table, should be peeled and neatly served. When they just crush between the hands, a secret worth knowing, they are cooked enough. Whole-meal bread, from wheat ground and prepared on the premises, and suffered to be at least two days old ere it be used, would prove a great economy. It is more highly azotized than, and, for common use, vastly preferable to white. Five ounces of it would be fully equal to, more than equal indeed, to six ounces of white bread. I consider the latter, as commonly made, a comparatively inferior food ingredient. Boiled barley, also boiled wheat or furmity, is very excellent food. The corn-heavers on the Vistula eat nothing else. It has been an English food from very remote antiquity. Beer, brewed on the premises, should be allowed daily or occasionally. Half a pint would not be too much. Tobacco, to many, would prove an immense boon. There are few things which the Irish insane poor, male and female, would not do—the one to obtain tobacco, the other tea. *Il va sans dire*, as the French say, that the food, under all circumstances, should be palatable, well-cooked, good of its kind, and neatly as well as attractively served. Insufficient or inferior nourishment, badly prepared and ill-served, is discreditable in itself, and hurtful to both body and mind in respect of those who, under whatever pretext, are condemned to partake of it.

*Belfast, March* 24, 1863.

